# Design and implementation of levee project information management system based on WebGIS

**DOI:** 10.1098/rsos.180625

**Published:** 2018-07-11

**Authors:** Bin Zhang, Yi-wei Ye, Xi-zhong Shen, Gang Mei, Han-xun Wang

**Affiliations:** 1 School of Engineering and Technology, China University of Geosciences (Beijing), Beijing 100083, China; 2 Key Laboratory of Deep Geodrilling Technology, Ministry of Land and Resources, Beijing 100083, China; 3 Yellow River Institute of Hydraulic Research, Zhenzhou, Henan 450003, China

**Keywords:** geotechnical information management system, levee project, WebGIS, browser/server mode

## Abstract

The China Levee Project Information Management System (CLPIMS) is an information management platform that was established for levee project management within the seven major river basins in China. The system was developed on the basis of the VS.NET and ArcGIS Server and was combined with the database theory and key techniques of WebGIS, which has the features of real-time display, enquiry, statistics and management of spatial data under browser/server mode. Moreover, additional applications, such as real-time monitoring, safety assessment, early warning and danger forecasting and online analysis, can be further explored through reserved modules. The CLPIMS can serve not only as a scientific, systematic, visual tool for analysis and decision management in levee projects in China but also as a technical platform for flood control practice. Furthermore, the system is capable of unified management and sharing of the levee project information for the seven major river basins in China, and it is important for the improvement of office automation, E-government applications and the level of flood control operations.

## 1. Introduction

**Figure 1.**
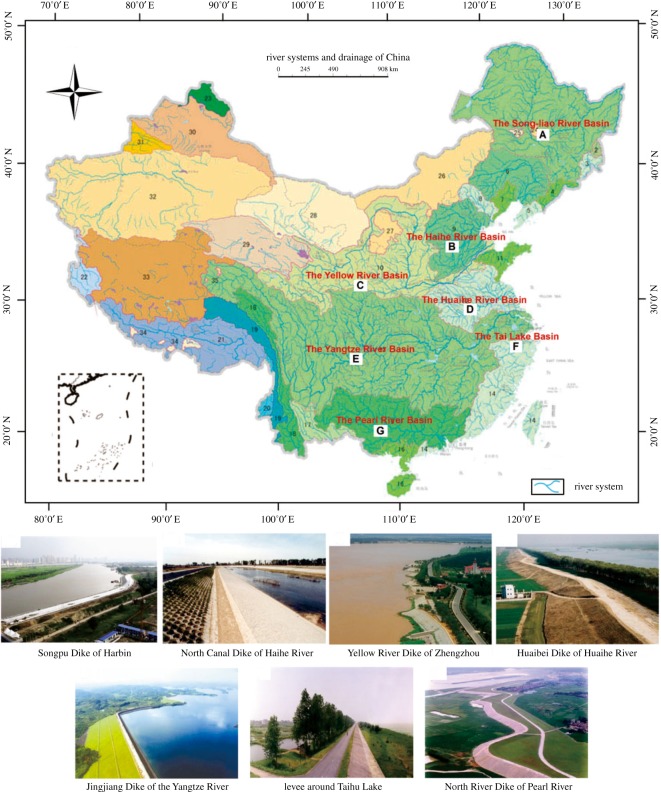
The seven major river systems and drainage in China.

With the improvement of human productivity and living standards, the development and management of water resources has become a major issue of global concern [1,2]. At present, there are more than 0.25 × 10^6^ km of various types of levee projects distributed in China's seven major river basins (figure 1), and many of these levees have potential safety problems [3–5]. To address the increasingly severe flood disasters and the safety status of levee projects, it is urgent to build an informational project management platform.

For the different demands of engineering projects, only few levee data of dangerous sections during historical hazardous events are available in the existing records and archives. The data are too fragmented and are unable to be updated and shared, reducing the efficiency of the construction and management of levees [6]. With the continuous development of information technology, geographical information system (GIS) has been widely used in levee data management because of its massive data storage and its powerful data management, analysis and graphical display functions [7–13].

In recent years, as Internet technology has been developing rapidly, GIS has gradually shifted from its traditional platform to a Web-based platform [14–21]. WebGIS (Web Geographical Information System) is a product combining a geographical information system and Internet technology, which has advantages such as distributed data management, independent platform, low system cost and efficient balancing of computational load. It provides a feasible technology for the development and sharing of geographical information [22–27]. In addition, Web-based GIS promotes the sharing and synthesis of multi-source data and can allow the spatial data model to be widely shared. Therefore, a management information system based on WebGIS can provide a powerful and effective way to manage levee information and make intelligent decisions regarding embankments [28–31].

Many decision support systems and information management systems have been developed to solve problems in river basin management. Oliveira *et al*. built a WebGIS observatory platform, tailored for risk assessment and emergency preparation and response in coastal areas [32]. In the USA, Deng *et al*. also developed a WebGIS-based decision support system (DSS) for managing recreational beaches [33]. The DSS consists of a sensor-assisted water quality monitoring system and a multiple linear regression model. The National Levee Database (NLD) was established by the United States Army Corps of Engineers (USACE). As of April 2012, the NLD included detailed information on more than 14 700 miles of levee systems that are associated with USACE programmes, but this is just a fraction of the total number of levees nationwide. The data of the NLD have been widely used in other studies [34]. Pradhan *et al*. developed an integrated disaster management system (DIORAMA) based on an enterprise system that supports GIS [35]. In France, Moins *et al*. developed the SIRS Digues software as a computing tool to make information on levees more durable and accessible in order to enhance their management [36]. The SIRS Digues relies on the document-oriented NoSQL database CouchDB and on geospatial libraries, namely, Geotoolkit and Apache-SIS. Simeoni *et al*. developed a system that combined WebGIS and a database management system [37]. The system was applied to the monitoring data of an embankment of the Adige River in northern Italy and was proved to be a suitable tool for the management and validation of real-time data and periodical field measurements. In China, similar information systems, such as the Pearl River Levee Project Construction and Management Information System and the Flood Control Information System of Dagu River Basin, were also developed [38,39]. However, these information systems are all regional and only contain levee project information on certain rivers. It is difficult for these independent information systems to share information with each other.

In this study, the China Levee Project Information Management System (CLPIMS) is developed. The system uses WebGIS as the core technology and adopts a multi-tier architecture based on the browser/server mode (B/S) [40]. Owing to the limitation of standard Structured Query Language (SQL) on spatial data processing, the system combines the SQL Server database and ArcSDE data storage model to achieve the fast storage of a large amount of complex types of data. A service system is established by applying Active Server Pages technology to achieve the cross-management of engineering data and to provide a platform for the digitization of levee structure [41]. The CLPIMS is a convenient, large-range information system. It meets the increasingly serious needs of levee project management in China, provides a platform for the collection and sharing of levee project information of different regions, and plays an important role in the flood prevention and control of levee projects.

## 2. Design of the CLPIMS

### 2.1. System development goals

The CLPIMS is a comprehensive system that is based on the data information of the seven river basins in China. The CLPIMS uses WebGIS as the core technology, combined with database and Internet technology. The purpose of the system is to provide scientific, systematic and visualized analysis and decision management tools for levee construction and projects management in China's seven major river basins and to provide a technical platform for flood prevention and mitigation.

### 2.2. System framework

**Figure 2.**
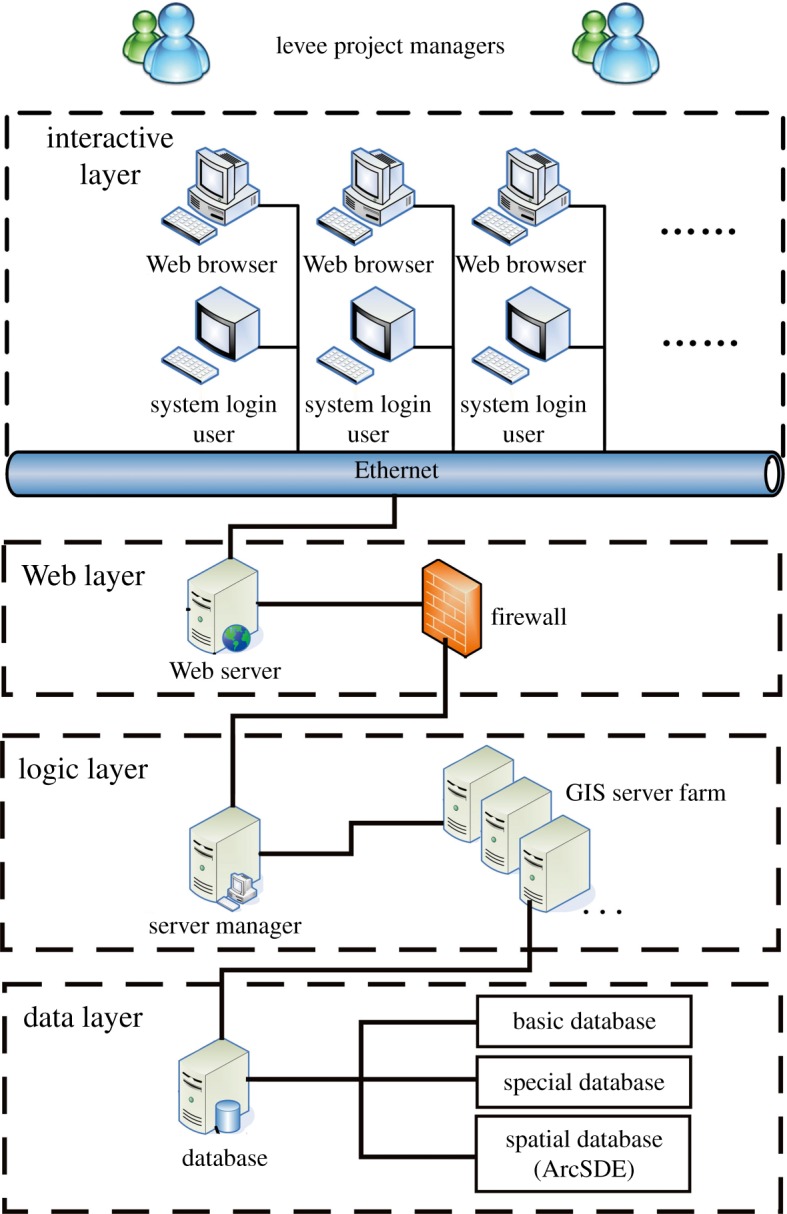
The logical structure of China Levee Project Information Management System.

The CLPIMS is mainly composed of four parts: the client terminal system, the database server, the appliance server and the network server. To simplify the system deployment and management in the design of the logical structure and centralize the management of data and services, a multi-tier architecture based on B/S was adopted in the system. The system can be divided into four layers, i.e. the interactive layer, Web layer, logic layer and data layer. The overall framework of the system is shown in figure 2.

#### 2.2.1. Interactive layer

**Figure 3.**
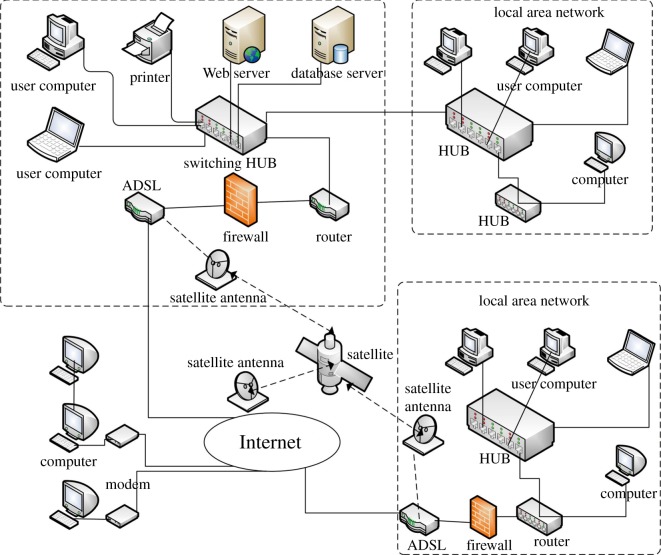
Schematic diagram of interactive layer network structure.

As a public interface for users to access the system, the main features of the interaction layer are the use of input devices or the Internet to send users' requests to the Web server through the Web browser or software system interface and display the feedback from the server to the users through output devices after a certain logic manipulation. The real logic processing is not implemented in this layer, as it only plays the role of forwarding users' requests. Figure 3 shows the schematic overview of the network structure.

#### 2.2.2. Web layer

The Web layer receives requests by users from the interactive layer and blocks illegal users' requests with a firewall to ensure the security of the system operation. A network application system developed using Asp.NET was deployed in the Web layer in order to carry out non-spatial data processing functions, except for GIS analysis. In addition, it also employs the AO component of the GIS server in the logic layer to complete the GIS processing function through remote object access technology.

#### 2.2.3. Logic layer

The logic layer is the centre of the GIS server, which is based on the ArcGIS Server platform. All internal GIS analysis and processing, including spatial data invocation, are implemented in the GIS server. Compared with other GIS platforms, ArcGIS has many technical advantages, especially in its object-oriented data model, long transaction management and version management. The Server Object Manager allocates complex and computationally expensive GIS operations to each server that is under its management through load balancing and computer cluster technology. The single server is only responsible for invoking the spatial data and the relevant operation, and then it returns the results to the Web layer.

#### 2.2.4. Data layer

The data layer is mainly responsible for the centralized management of all data in the CLPIMS. The spatial data are stored in the data centre in the form of a spatial database. The spatial data are indexed and described through a spatial information metabase, while the attribute data are distributed among various departments, and the centralized management and seamless sharing of the distributed data are implemented by establishing the metadata of the attribute data.

In summary, the data layer operates on the database and provides data services for the logical layer; the logical layer addresses the business logic processing of the data provided by the data layer and sends it to the interaction layer through the Web layer; the Web layer accepts and passes the request of the interaction layer and the feedback of the logic layer and ensures the security of the system through the firewall; and the interaction layer directly accepts the user's request and displays the results.

In the development of the system, different layers are designed to be as independent as possible. Compared to other structures, this multi-tier architecture has some obvious advantages. The structure of hierarchy and division of labour are clearer, different functions are realized at different layers, and the developers can focus on one of the layers at a time. This could well use the system resource. Moreover, the system is easy to maintain and update, which improves the system stability. It also reduces the bottleneck effect on the system performance caused by the large and complex GIS data operations and increases the processing ability of the system to handle the large number of concurrent users accessing the system [42,43].

The multi-tier architecture also makes the system highly portable. The interactive layer can be replaced as one part to transplant the middle layers to other new similar systems. In addition, because all the major relational database management systems support the SQL language, the programs written in SQL are also highly portable. At the same time, this system is designed for levee projects in seven major river basins nationwide; the scope is large. With the online operation of the system, it will inevitably involve the addition and alteration of the levee project information. The addition of projects under such a structure mainly relates to the underlying data layer changes, which is relatively easy to implement. However, a possible problem is that with the increase in the amount of system data, the efficiency of the system will be affected.

## 3. Implementation of the CLPIMS

### 3.1. System data

The system makes full use of the advantages of GIS in spatial data storage and analysis and has completed the integration and processing of basic data for a number of Chinese levee projects over the years. It satisfies the requirements of information sharing and reduces repeat work, thus greatly improving the efficiency of the water conservancy administrative departments in water conservancy project construction. The basic data of the levee projects are the core when developing the information management system. In addition, the system also provides a basic guarantee for the informational and systematic management of the levee projects.

#### 3.1.1. Data range

This study geographically involves the seven major river basins in China, including the Yangtze River Basin, the Yellow River Basin, the Huaihe Basin, the Pearl River Basin, the Songliao Basin, the Haihe River Basin and the Taihu Lake Basin. The distribution of the above seven basins is shown in figure 1. The main contents of the database include the following:

(a) General overview of the levee projects in the seven basins.
(b) Character of the main stream.
(c) Geology of the river basins.
(d) Major engineering geological problems.
(e) Engineering geological conditions of the important levees.
(f) Engineering geological conditions of the endangered levee sections.

#### 3.1.2. Data sources

All data were obtained from levee project management agencies with independent legal personnel through a comprehensive survey.

The typical project information collected in the survey mainly includes the project overview, the comprehensive information, risk and danger statistics, engineering geology, engineering profiles and culverts. The basic information of the river basins and levee projects was provided by the competent authorities, digital information development and management departments and project management departments, while the dedicated data were mainly provided by project management departments and engineering design departments. With these data of typical levee sections, the CLPIMS can provide technical support for levee construction, management and scientific research.

#### 3.1.3. Selection of typical levee projects

According to the requirements of database design and system development, the engineering data of the typical levee projects of the seven major river basins in China were selected as the basic data of the system. The typical levee projects selected meet the following conditions:

(a) Levee projects that belong to the first, second or third levels of the national embankment standard.
(b) Levee projects that have complete basic data and a certain amount of dedicated data, including the physical and mechanical properties of the soil and the permeability coefficient.
(c) Levee projects with potential hazards.
(d) The typical length of the selected levee segments was approximately 10 km.

Table 1 shows the general overviews of the typical levee projects of the seven major river basins.

**Table 1.** The typical levee projects of the seven basins.

basin	levee project	mile point	length (km)	project class	general situation of dangers
Yangtze River	Jinjiang	729 + 000 to 741 + 288	12.29	I	mile point 740 + 342 to 741 + 288 in Guanyinsi section, more than 5000 dangers occurred in 1954, and during the 1987 flood, a major danger occurred in the foundation of the levee
Yellow River	Zhengzhou huanghe	0 + 000 to 70 + 250	71.42	I	14 flood hazards occurred from 1919 to 1928
Huaihe River	Huaibei	33 + 400 to 47 + 700	14.30	I	levee breached in mile point 33 + 400 to 36 + 035 in Raojin section during flood season in 1954, and serious seepage occurred during flood season in 1991 and 1996
Haihe River	Zhangwei xinhe	42 + 500 to 79 + 000	36.50	II	mile point 42 + 500 to 79 + 000 section is of relatively poor condition, prone to danger
Pearl River	Beijiang	6 + 750 to 28 + 650	21.90	I	6 + 750 to 28 + 650 section is a historical dangerous levee section, several major dangers occurred during the floods in June 1994 and July 1997
Song-liao River	Harbin urban Jiangbeisongpu	0 + 000 to 8 + 850	6.85	I	seepage and scour of levee occurred in the 1998 flood in mile point 1 + 000 to 3 + 950 section
Tai Lake	Tai Lake levee wujiang section	CS26 + 500 to CS50 + 367	23.87	II	several dangers occurred in the Tai Lake levee wujiang section during the 1999 flood

### 3.2. Database building

#### 3.2.1. Database structure

##### 3.2.1.1. Database category

**Figure 4.**
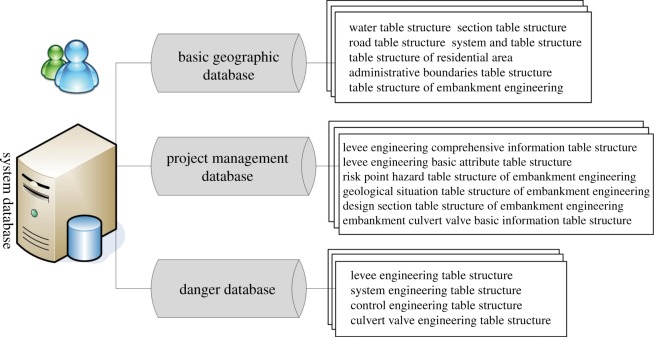
The category of database.

The CLPIMS database information includes the following: historical data, real-time data, project information, hydrological data, water environment data, GIS data, disaster information, meteorological data, multimedia data, flood and disaster control information and DEM data [44]. To facilitate the effective organization and management of massive amounts of information in the synthesis database, the synthesis database was divided into a basic geographical database, project management database and danger database. Table 2 lists the typical information of the levee projects in the CLPIMS. Figure 4 shows the category of the databases.

**Table 2.** Typical levee project information data table.

no.	field name	field identifier	type and length	unit	nullable
1	name	Name	nvarchar(20)	/	no
2	river name	Beloriv	nvarchar(20)	/	yes
3	river information	RiverInfor	nvarchar(40)	/	yes
4	tributary information	TribInfor	nvarchar(20)	/	yes
5	levee length	Bnscln	nvarchar(50)	km	yes
6	levee starting pile number	Stpegno	nvarchar(12)	/	yes
7	levee terminal pile number	Endpehno	nvarchar(12)	/	yes
8	dike type	DikeType	nvarchar(12)	/	yes
9	levee starting position	Stapla	nvarchar(12)	/	yes
10	levee terminal position	Endpla	nvarchar(12)	/	yes
…..	…..	…..	…..		

##### 3.2.1.2. Database logic structure

The overall design logic of the database is to determine the organizational forms of mass data from the perspective of the user of the database [45,46]. The data organization of the CLPIMS is planned and designed using the method of ‘spatial partitioning and layering based on thematic’. The database is divided into seven parts by space, i.e. seven major river basins. Each basin is independent as a complete Geodatabase, such as the Yellow River Basin Geodatabase. Each Geodatabase is divided into different Feature Datasets according to different thematic aspects. The Feature Dataset stores the vector map layer data of different thematic aspects, i.e. different Feature Classes. For example, the Engineering Feature Dataset stores the levee Project Distribution Map Layer, Drainage Pumping Station Distribution Map Layer, etc. All Feature Datasets are organized as sub-databases, such as the Basic Geographical Database, Hydrological Database, Meteorological Database, Engineering Database, Population, Society and Economy Database, Land Utilization Database and Relational Information Database. All data tables were designed in strict accordance with the relevant national standards and norms to enable sharing and efficient use of the data. Different spatial data were expressed by different spatial indices, and spatial metadata can be used as a separate logical unit, which can describe and manage the whole spatial database.

#### 3.2.2. Data organization and management

**Figure 5.**
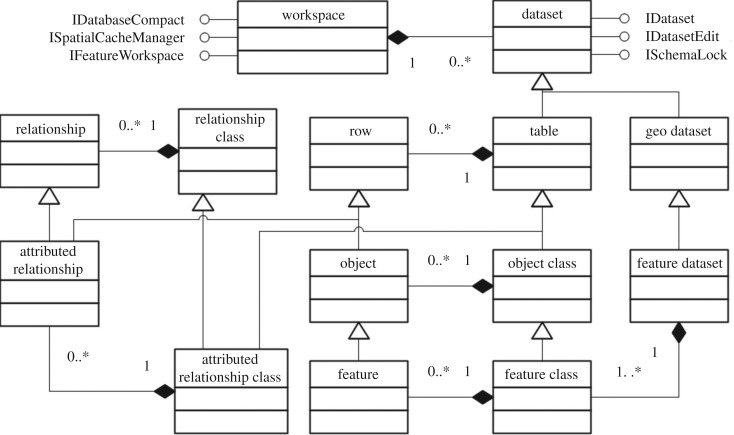
Geodatabase data model.

The database is not only the foundation of the geographical information system but also the foundation of the CLPIMS. The data quality strongly affects the accuracy of the whole system analysis directly, and the rationality of its data structure directly affects the maintenance and update process of the entire operating system. Therefore, the construction of the database was in strict accordance with the process of data requirement analysis, database design, data integration and input, quality control and data integration technology. The CLPIMS database adopts the Geodatabase data model to organize data, and figure 5 shows a simplified view of the objects in the Geodatabase model. To facilitate the design, all of the attribute data exist as associated tables rather than being put directly into the attribute tables of the spatial data. Only space items are stored in the attribute table of the spatial data; other attributes are stored in the associated table. Data can be imported into the database in two ways, i.e. manual input and file import. Manual input is only suitable when there is a small amount of data, but in most cases, there is a large amount of data, and the data can only be directly imported through the transformation of Excel file data with a specific format to SQL data. Users can download the specified Excel data template file through the Web server and then import it into the database after local input.

— A workspace in the Geodatabase data model corresponds to a Geodatabase.
— A dataset is the highest level container of data.
— A geodataset is a dataset that contains geographical data.
— A feature dataset is composed of feature classes, geometric networks and topologies.
— A table is a collection of rows that have attributes stored in columns.
— A row is a record in a table. All rows in a table share the same set of fields.
— An object class is a type of table that stores non-spatial objects.
— An object is a row with an object identifier.
— A feature class is a type of object class that stores spatial objects.
— A feature is an object with a geometric shape.
— A relationship class represents relationships through embedded foreign keys.
— A relationship is an association between objects or features and controls behaviour when objects or features are moved or deleted.
— An attributed relationship class is a type of table that stores relationships.
— An attributed relationship can represent many-to-many relationships and attributes on relationships.

##### 3.2.2.1. Attribute data organization and management

Project management data, such as the levels and types of levees and other levee profile basic attributes, have to be shared with superiors, subordinates and organizations from other basins; therefore, the database needs to be designed in accordance with the National Hydrological Information Database Standard.

Attribute information that only relates to spatial information, such as the levee project name and the longitude and latitude of the levee projects, was associated with the spatial information layer, using the space object ID as the primary key. Some of the other simple attributes, such as the name, can be directly stored as extended attributes of the spatial information layer.

##### 3.2.2.2. Spatial data organization and management

Different from the common database system, the data in the levee project information management system database should be organized on the basis of the spatial location, which is mainly reflected in the GIS data. The two were connected through the geographical object ID, thus forming a complete spatio-temporal database [47]. Therefore, GIS data can be considered as the core of the levee project information management system. The hierarchical organization and coding of GIS spatial objects has become the de facto standard of the GIS data organization form. We can also organize the GIS data into layers of administrative divisions, transportation, geology, topography, geomorphology, soil types and distribution, land use, hydrology, engineering, etc. A layer is a relationship table, taking the ID of the spatial object as the primary key, while the table includes a field that stores space information (generally named *Shape*); each record in the table represents a spatial object. From a physical point of view, the traditional GIS spatial data are commonly managed using the file format, such as Coverage, Shape of ESRI and Mif of MapInfo, which are simple and easy to implement but do not support the concurrent response.

## 4. Results

**Figure 6.**
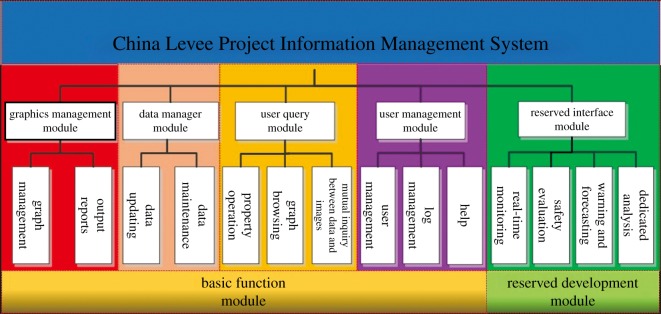
Schematic diagram of the components of the system module.

**Figure 7.**
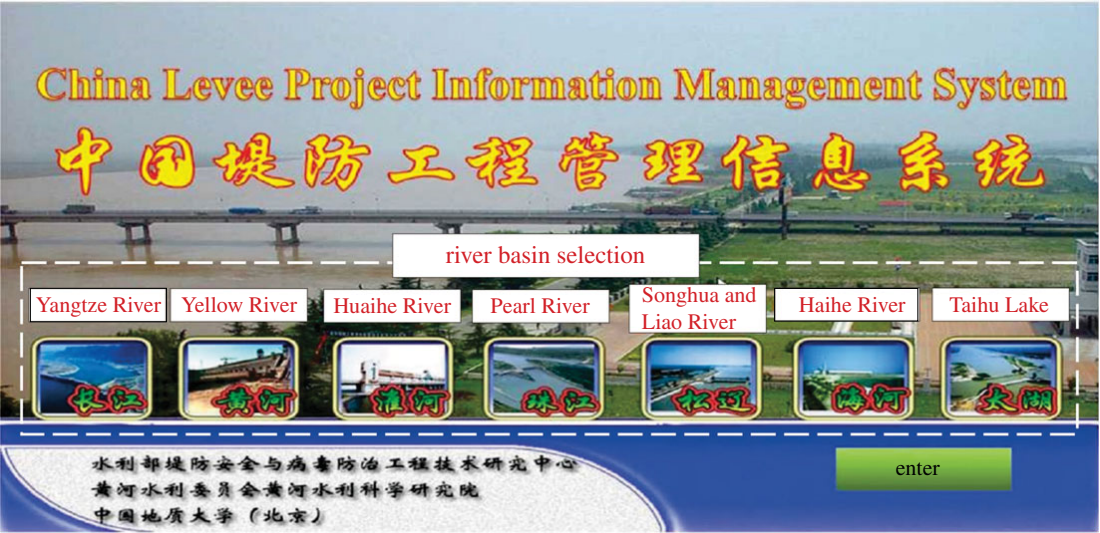
System main interface.

The CLPIMS fully used the advantages of GIS in spatial data storage and analysis. It integrated and processed the data of the seven major river basins across the country over years to meet the requirements of information sharing. The system consists of eight application modules, including four basic functional modules (i.e. the graphics management module, data management module, user query module, and user management module) and four reserved modules (i.e. the real-time monitoring module, safety evaluation module, warning and forecasting module and dedicated analysis module) [48–50]. The four reserved modules are not fully developed, but the system has module interface set-up. Through the different features of the above modules, users can browse and manage the levee data quickly and with flexibility. The system supports water conservancy technical personnel with levee information management, improving efficiency. Through the dynamic updating of the basic data, information services are provided for flood control and mitigation. By interfacing with the four reserved modules or other specialized analysis system, the CLPIMS can provide real-time safety monitoring, online analysis and early warning, which may provide technical support for flood control and mitigation. Figure 6 shows the schematic diagram of the system module components, and figure 7 shows the main system interface. In the main interface, there are seven bottoms representing the seven major river basins in China, and users can choose to enter the subsystems of different basins and carry out information management operations.

### 4.1. Graphics management and report output

**Figure 8.**
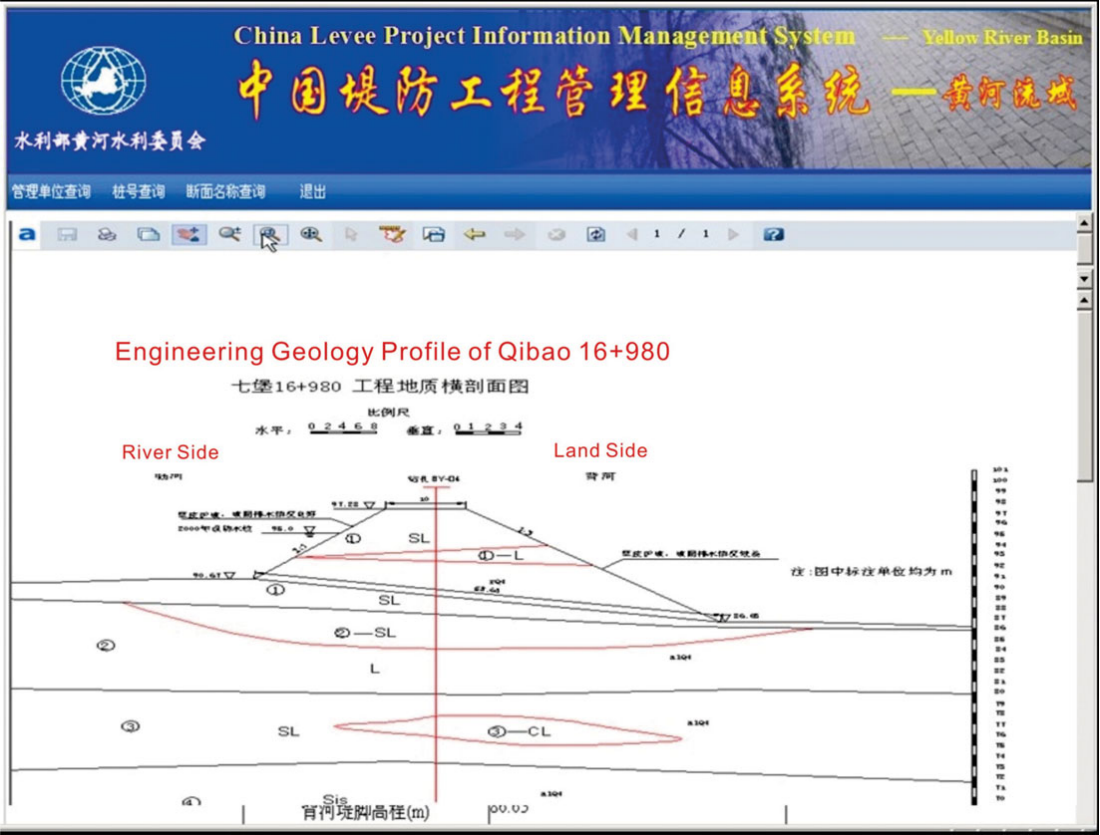
Levee profile output interface.

With the support of the integrated database and GIS, the CLPIMS can display the distribution, grade, type, historical accidents, drill column, vertical and cross-section geological data, current situation and other basic information and statistical results of the levee project in each river basin in simple and intuitive forms, namely, charts and tables. CLPIMS mainly produced output functionality in the form of graphics, text and report forms. According to the requirement of the spatial pattern distribution and display of the construction and demolition of the levee project, a graphic management subsystem is developed based on ArcGIS. Figure 8 shows the output interface of a profile named ‘Qibao’ in a typical levee project. In this interface, users can browse the professional information on levees, such as the engineering geology profile, in the system database and facilitate the management of the levees.

### 4.2. Data management and update

**Figure 9.**
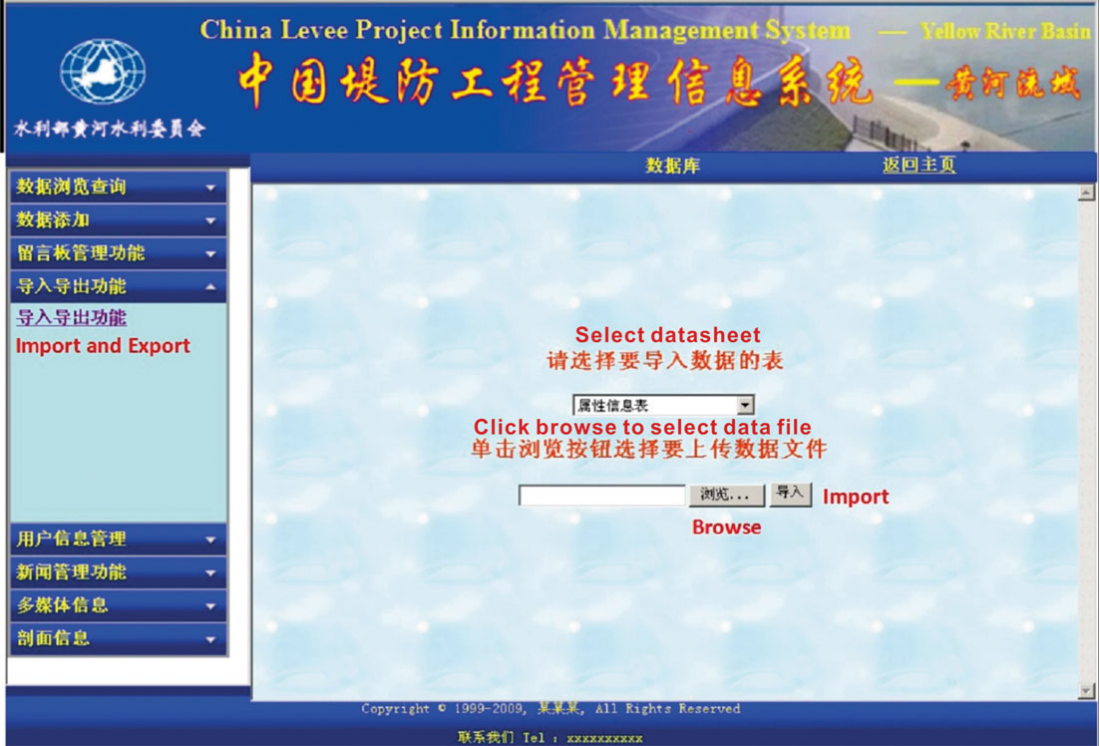
System data import interface.

**Figure 10.**
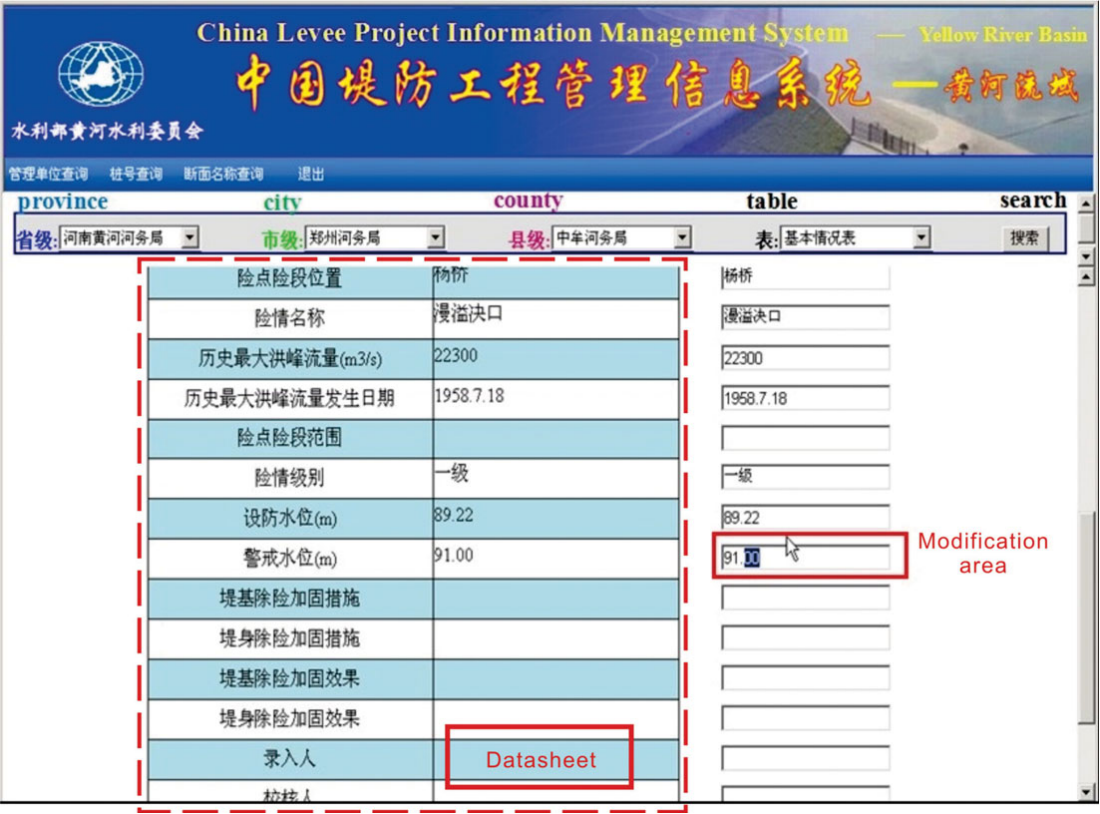
System data modify interface.

Based on the established spatial database and attribute database, the CLPIMS can input, delete and update information through the network transmission (Web) in a variety of data formats (Excel, Dat, etc.), which thus enables the real-time update and management of the database. Users with different permissions can perform corresponding operations on the data in the database through the browser, including browsing, adding, deleting and modifying data. It is convenient for levee administrators to report information in a timely manner, maintaining the real-time and accuracy of the system data, strengthening the connection between different levee sections, heightening working efficiency and ensuring embankment safety. In addition, in cases of temporary network outage or other situations where a network cannot be connected, users with different permissions can continue updating and maintaining the data through the computer software terminals; once the network is recovered, updates can be released immediately, thus improving the ability of the system to address unexpected problems. Moreover, the system database maintenance module also provides a dedicated tool kit that can perform the initialization, sorting, backup and recovery of the database. Figure 9 shows the system data import interface. In this interface, users can click the ‘Browse’ button to browse local files to import or to export online files. Figure 10 shows the system data modify interface. In this interface, users can choose specific levee project information datasheets and modify the outdated information.

### 4.3. Information search and retrieval

**Figure 11.**
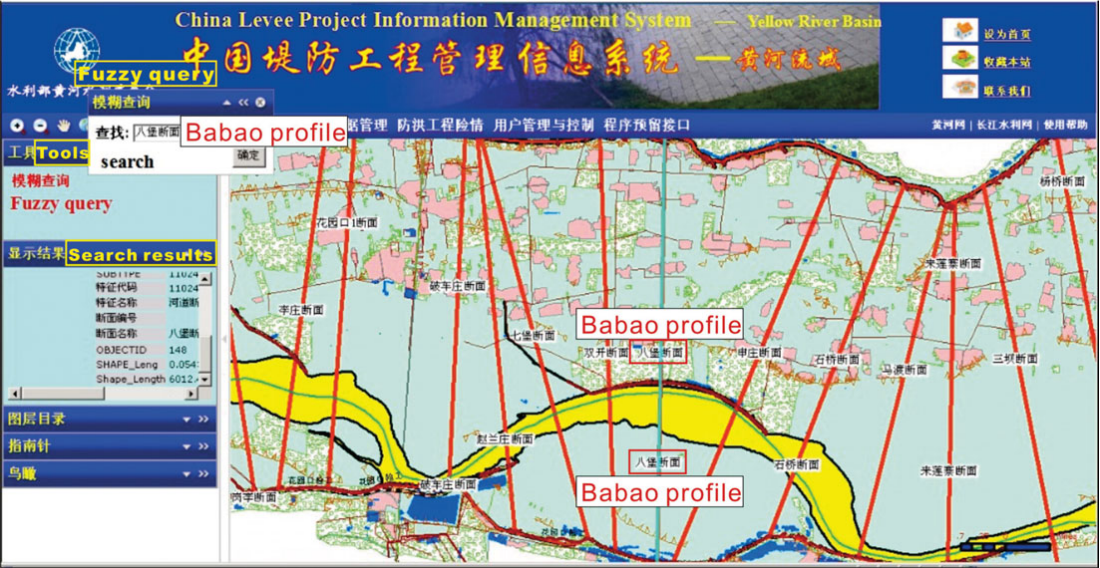
System information fuzzy query and retrieval interface.

**Figure 12.**
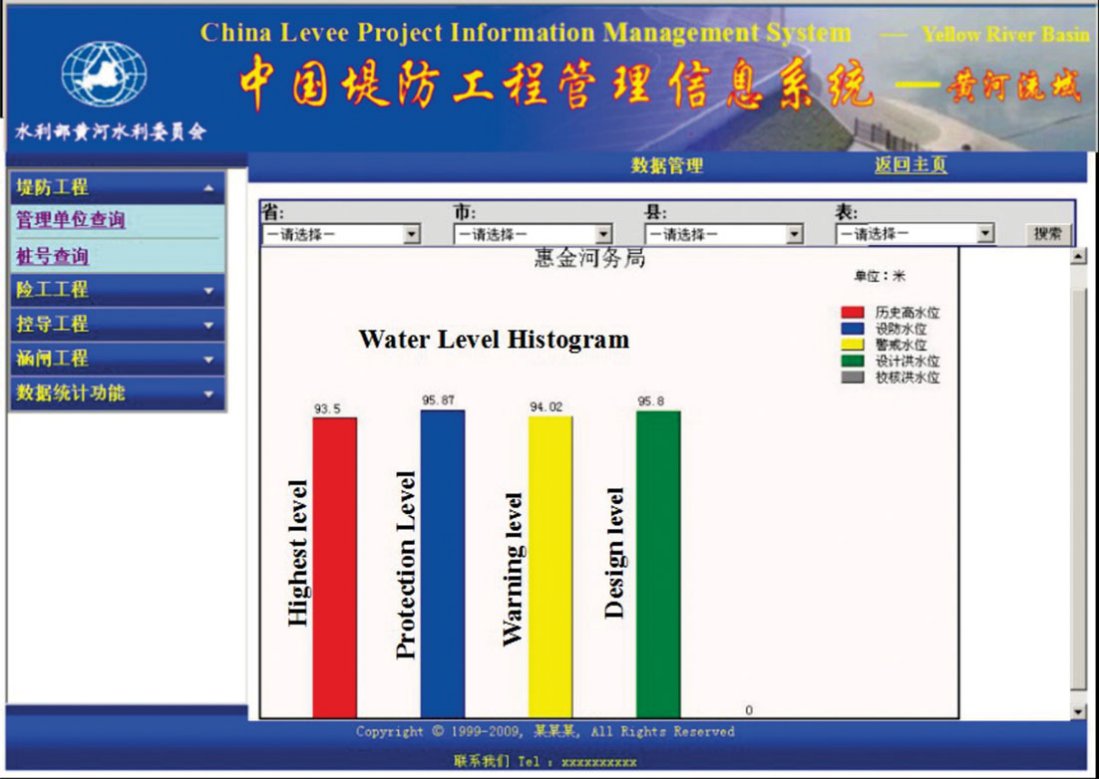
System statistical chart interface.

The system uses the GIS as a platform and the integrated database as the data storage system. Through the fuzzy query, the retrieval of the point, line and polygon information, and the data display in the forms of graphics, text, statistical charts, etc., the system basically provides full access to all kinds of information on the different types and different levels of levees in each river basin. The required data can be obtained by querying the field in different methods, and the query results can be applied to generate histograms, line charts, pie charts, radar images and continuous curves according to the needs of the user. The generated charts can be saved to a local disc. Figure 11 shows the system information fuzzy query and retrieval interface. Fuzzy query can provide the results of the synonyms of keyword searches, which can improve the search efficiency when the specific keyword is not determined. In this interface, the search keywords are ‘Babao profile’, and the qualified result is shown in the search result column, while the location of the profile is shown in the map. Figure 12 shows the system statistical chart interface; this interface can provide different types of statistical charts according to the needs of the user.

### 4.4. User management and access control

All the seven major river basins were under the management of the Ministry of Water Resources, yet every river basin has its own competent department, i.e. Water Conservancy Commission. Therefore, the system has many potential users, and users from different departments have different responsibilities. To ensure the security and integrity of the database, the system needs to grant different users corresponding access according to the situation.

**Figure 13.**
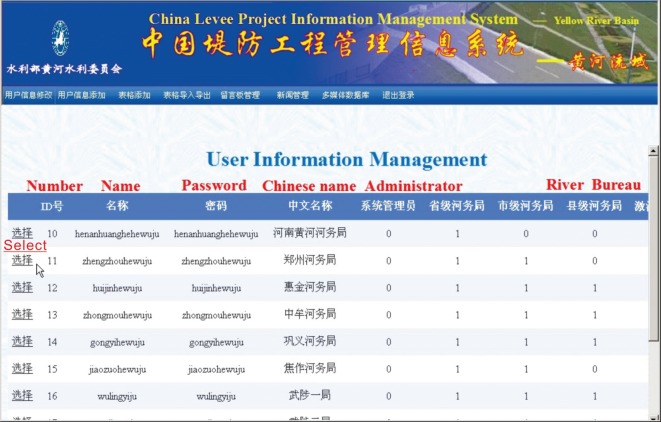
User information management interface.

The users of this system are mainly production technicians, researchers, managers and leaders from design or construction departments of all levels of levee projects nationwide. This system adopts data item values and given special modules to allow access control. Before entering the system, it uses the ‘user identifier’, i.e. the username to identify the users, and uses the password to confirm the users; thus, the system can review the user's permissions [51]. In the application design, IP addresses and cookies are combined to ensure that users at all levels can only access pages under permission. Furthermore, Security Socket Layer encryption technology is adopted at the Webserver end to ensure information interaction security between the client and site. By adopting network segment isolation technology, most important hosts and intranets are physically isolated from the Internet. Thus, the security and confidentiality of the system are guaranteed. Figure 13 shows the system user information management interface; in this interface, the system administrators can modify the user permissions.

## 5. Discussion

In this study, there are four reserved module interfaces in the CLPIMS, i.e. the real-time monitoring module, safety evaluation module, warning and forecasting module and dedicated analysis module. Although these modules are not implemented, they are still important. The most important role of levee projects is the prevention and mitigation of flood hazards, and a reasonable early warning and decision-making mechanism can effectively prevent potential disasters or reduce the loss to a minimum [52,53]. Therefore, the purpose of the four reserved modules is to help build a rapid and effective early warning mechanism.

Real-time monitoring is essential in an early warning system but requires a whole set of tools for collecting, managing and analysing a large amount of geospatial and temporal data, including water level, land subsidence, surface and soil displacements and pore-water pressure. In cases of the sudden outburst of flood hazards, some of these indicators may change rapidly; thus, these measurements should be collected automatically at high frequencies. This requires the deployment of sensors in historical high-risk levee sections to monitor the abovementioned indicators. In addition, monitoring data will be uploaded to the CLPIMS and stored in a special database for real-time monitoring. According to the pre-stored data in the system database and the information accessed from the real-time monitoring module, a safety evaluation can be achieved in the safety evaluation module. The dedicated analysis module mainly addresses the engineering geological condition data of key sections of the levee projects, the design and construction data, seepage and slope stability evaluation data and monitoring point layout data. Using the pre-stored data in the system database and the information accessed from real-time monitoring, as well as data uploaded by levee project management personnel, the analysis of piping, soil flow, embankment collapse and other dangers as well as their most likely location could be realized. Finally, administrators can publish early warning information through the warning and forecasting module.

Another important function of this system is online analysis. Combined with these four modules, online analysis can be used to perform safety evaluation and dedicated analysis or other computations with a large amount of data, such as the assessment of some non-emergency safety problems. In this system, GIS components can be distributed in several GIS servers with distributed object technology; thus, some functions in the GIS components are called through Remote Procedure Call and message passing. Hence, GIS function processing and non-GIS function processing are separated, and the analysis is implemented online in the server end, reducing the load of the client program or the Web application server in processing the GIS functions.

Through the development of this information management system, levee project information can be centrally managed in real time from different regions of China, and we can improve the work efficiency of project management personnel and reduce the duplication of effort. However, there is a potential problem in that, due to the new construction of the system, there are no sufficient data other than those of typical levee projects. Compared to the information systems in other studies, the CLPIMS developed in this study covers the main river basins across the whole country, containing more information. With the operation and use of the system through the years, the database will become larger and more mature as more data are uploaded. It might be a problem to run online analysis with a large amount of data. Therefore, the system operation efficiency needs to be improved in future studies.

## 6. Conclusion

To meet the demand of flood control, water resource allocation, levee project management and levee information sharing in China, the CLPIMS was developed. The system uses the VS.NET and ArcGIS Server technology as the platform, combined with the key technology of databases and WebGIS. The system facilitates the visualization and intelligent design of levee project information management, facilitating levee project management in China.

The river basins in China are managed by different departments or river bureaus, and information sharing was inconvenient. The CLPIMS covers the seven major river basins in China and facilitates the interactive sharing of levee information. By logging into the information management system, levee management personnel from different river basins and different organizations can upload levee information in a timely manner. Thus, this can standardize the levee project information management in these river basins and facilitate the real-time sharing and centralized management of levee project information, changing the previous situation of information sharing hysteresis and management discrepancy.

The CLPIMS also attempts to integrate the early warning system. The system contains four basic modules and four expansion modules. With the full development of these features and the use of the data from the CLPIMS database, management personnel can perform safety assessments and early warnings, providing technical support for levee project construction and flood control.

## Data accessibility

In addition to the terrain information, engineering geological characters and other data of the seven major river basins in China included in the system, there are no experimental data in this paper.
